# Neues Zulassungsverfahren Humanmedizin: höhere individuelle Gerechtigkeit, aber Verstärkung des Landarztmangels?

**DOI:** 10.1007/s00103-023-03825-x

**Published:** 2024-01-10

**Authors:** Brigitte Müller-Hilke, Claudia Finger, Wolfgang Hampe

**Affiliations:** 1 Core Facility für Zellsortierung und Zellanalyse & Institut für Immunologie, Universitätsmedizin Rostock, Schillingallee 70, 18057 Rostock, Deutschland; 2 Wissenschaftszentrum Berlin für Sozialforschung, Berlin, Deutschland; 3 Zentrum für Experimentelle Medizin, Institut für Biochemie und Molekulare Zellbiologie, Universitätsklinikum Hamburg-Eppendorf, Hamburg, Deutschland

**Keywords:** Wartezeitquote, Zusätzliche Eignungsquote, Abiturnotenausgleich, Ärzt:innenmangel, Landarztquote, Waiting quota, Personal suitability quota, Compensation for GPA, Shortage of rural doctors, Rural doctors quota

## Abstract

**Hintergrund:**

Das Bundesverfassungsgericht hat 2017 die Wartezeitquote und die Beschränkung der Ortspräferenzen bei der Vergabe der Studienplätze in der Humanmedizin als verfassungswidrig erklärt und einen Bundeslandausgleich für Abiturnoten gefordert. Daraufhin wurde ab 2020 die Wartezeitquote durch die „Zusätzliche Eignungsquote“ ersetzt, die Zahl der Ortspräferenzen nicht mehr begrenzt und ein Abiturnotenausgleich eingeführt. Die vorliegende Studie untersucht, welche Auswirkungen diese Umstellung auf die neuen Erstsemester hat.

**Methoden:**

Daten der Stiftung für Hochschulzulassung wurden für die letzten beiden Wintersemester (WS) vor und die ersten 3 WS nach der Umstellung verglichen.

**Ergebnisse und Diskussion:**

Während sich der Einfluss des neuen Verfahrens auf die Studierenden mit vorheriger medizinnaher Ausbildung noch nicht endgültig beurteilen lässt, bleiben durchschnittliche Abiturnote und Frauenanteile annähernd unverändert und der Studienort ist nach wie vor bevorzugt wohnortsnah. Die Studierenden sind jünger geworden und der Länderausgleich gleicht die Chancen für Abiturient:innen aus Bundesländern mit besseren und schlechteren Abiturnoten an. Ein neues Ungleichgewicht entsteht jedoch, weil der Länderausgleich die Anzahl der Bewerber:innen berücksichtigt – und ländlich geprägte Bundesländer weniger Bewerber:innen hervorbringen. Da aber Landärzt:innen häufig auch ursprünglich aus ländlichen Gebieten stammen, ist eine Verschärfung des Ärzt:innenmangels gerade in den neuen Bundesländern ein mögliches Zukunftsszenario. Ein veränderter Notenausgleichsmechanismus könnte hier zusätzlich zur Landarztquote entgegenwirken.

## Abstract (en)

**Background:**

In 2017, the Federal Constitutional Court ruled several aspects of the German student admission process unconstitutional. Consequently, the waiting time quota was replaced by an aptitude quota in 2020. Students are now allowed to simultaneously apply to all German medical faculties and an adjustment algorithm for school leaving grades from different federal states was introduced. The present study investigates the impact of these changes on the new study cohorts.

**Methods:**

Records from the federal admission trust were used to compare the final two winter semesters before the change to the first three thereafter.

**Results and discussion:**

The impact of the new procedure on students with previous medical training cannot yet be conclusively assessed. While grade point average (GPA) and sex of the students remained comparable and students still prefer to study close to home; however, they have become younger. The adjustment for school leaving grades indeed led to equal opportunities for the individual applicant; however, this may aggravate the shortage of rural doctors. The current adjustment mechanism considers applicant numbers, yet less people apply from rural areas while at the same time these areas suffer from a shortage of physicians. As rural upbringing and education are the best predictors of rural practice after licensing, the shortage may worsen. To counteract this, the compensation mechanism for the school leaving grades could easily be adjusted.

## Einleitung

Im Urteil des Bundesverfassungsgerichts vom Dezember 2017 [1] wurden wesentliche Aspekte der Medizinstudierendenauswahl für verfassungswidrig erklärt. Daraufhin reformierten die Bundesländer die Auswahlregeln mit einem Staatsvertrag [2], wobei

1. in den Auswahlverfahren der Hochschule (AdH) die Note der Hochschulzugangsberechtigung (HZB) durch ein weiteres Kriterium und einen Studieneignungstest ergänzt werden muss,
2. ein Ausgleich der HZB-Noten aus unterschiedlichen Bundesländern auch im AdH erfolgt,
3. bei der Bewerbung jetzt mehr als 6 Studienortswünsche (Ortspräferenzen) angegeben werden und die Fakultäten diese Ortspräferenz nicht mehr als Vorauswahlkriterium verwenden dürfen,
4. die Wartezeitquote (nach einer Übergangsfrist) abgeschafft und durch die Zusätzliche Eignungsquote (ZEQ) ersetzt wird, in der die HZB-Noten nicht als Auswahlkriterium herangezogen werden dürfen.

**Figure 1:**
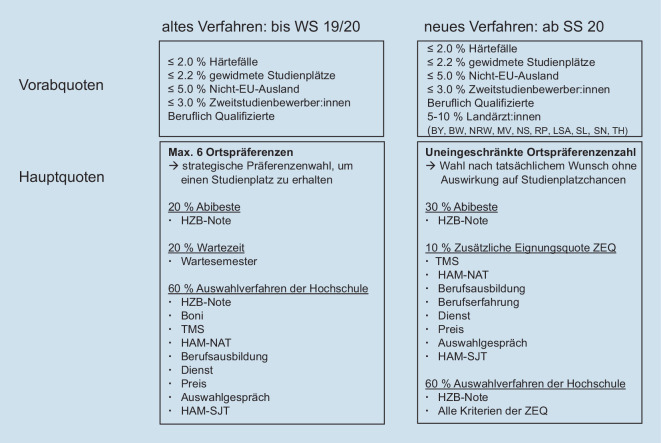

Die neuen Regelungen traten zum Sommersemester (SS) 2020 in Kraft und wurden durch die Fakultäten und die Stiftung für Hochschulzulassung (SfH) umgesetzt. Die Studienplätze jeder Fakultät werden seitdem nach dem Abzug von einigen Vorabquoten in der Hauptquote zu 30 % an die Abiturbesten vergeben (Abb. 1). Im AdH (60 %) und in der ZEQ (10 %) müssen die Fakultäten 2 zusätzliche Auswahlkriterien wählen, wobei die HZB-Note in der ZEQ keinen Eingang finden darf, im AdH aber als zusätzliches Kriterium gewählt werden muss.

Weitere Kriterien, neben den Studieneignungstests für Medizinische Studiengänge (TMS), Hamburger Naturwissenschaftstest (HAM-NAT) und Hamburger Situational Judgement Test (HAM-SJT), sind Berufsausbildung bzw. -erfahrung, Dienste, Preise und Auswahlgespräche [3]. In einer Übergangsregelung wurde die Wartezeit bis zum Wintersemester (WS) 2021/2022 noch als hoch gewichtetes Kriterium angerechnet.

Etwa zeitgleich mit der Umstellung der Studierendenauswahl auf das neue Verfahren haben einzelne Bundesländer – Nordrhein-Westfalen (NRW) war hier Vorreiter – damit begonnen, zwischen 5 % und 10 % ihrer zu vergebenden Studienplätze in einer Vorabquote der Ausbildung zum/zur Landärzt:in zu widmen [4].

In der vorliegenden Studie wird unter Zuhilfenahme der SfH-Datenbanken untersucht, wie sich das veränderte Vergabeverfahren auf die neu Eingeschriebenen auswirkt: Haben sich die Alters- oder Geschlechterverteilungen verändert? Hat der Anteil der Studierenden mit vorheriger medizinnaher Ausbildung zugenommen? Wie verteilen sich die Studierenden über die Republik nach Wegfall der eingeschränkten Ortspräferenz? Und wie verteilen sich die Abiturnoten auf die mittlerweile 39 Fakultäten?

## Methoden

Die dieser Studie zugrunde liegenden Daten stammen aus den Datenbanken der SfH und beziehen sich auf Bewerber:innen und die eingeschriebenen Studierenden. Eine geringfügige Unschärfe in den Daten resultiert daraus, dass die Fakultäten ihre finalen Einschreibungen nur unvollständig zurückmelden. Somit blieben zwischen 10 % und 15 % der Einschreibungen hier unberücksichtigt.

Weil nicht alle Fakultäten zum SS einschreiben, wurden in der vorliegenden Studie die WS 2018/2019 und 2019/2020 für das alte und die WS 2020/2021, 2021/2022 und 2022/2023 für das neue Verfahren berücksichtigt. Dabei gilt zu beachten, dass als Übergangsregelung für die Jahrgänge 2020/2021 und 2021/2022 die Wartezeit noch mit 45/100 bzw. 30/100 Punkten in der ZEQ berücksichtigt wurde, bevor sie im WS 2022/2023 für die Zulassung zum Studium meist bedeutungslos wurde.

Um die Verteilung der HZB-Noten an den Fakultäten zu untersuchen, wurde mangels ausreichender Differenzierung der Durchschnittsnoten das Punktesystem für Abiturnoten genutzt. Dafür wurden für alle Eingeschriebenen die jeweiligen Punkte in Prozent der maximal möglichen Punktzahl (840 oder 900) umgerechnet. Der Vergleich der Jahrgänge untereinander erfolgte mittels Kruskal-Wallis- und anschließenden Post-hoc-Tests.

Um zu untersuchen, wie viele der zur Verfügung stehenden Studienplätze an die 16 Bundesländer vergeben wurden und wie sich die Studierenden dann über die Republik verteilten, wurden neben den Zahlen der SfH zu Bewerber:innen und Eingeschriebenen die innerdeutschen Wohnorte bei Bewerbung sowie Daten des Statistischen Bundesamtes [5] genutzt. Die mittleren HZB-Noten für jedes Bundesland entstammen der Statistik der Kultusministerkonferenz [6], die Dichte der Ärzt:innen der Ärztestatistik 2021 der Bundesärztekammer (Stichtag 31.12.2021; [7]). Für den Vergleich der Eingeschriebenen nach altem und neuem Verfahren wurden die Werte der WS 2018/2019 und 2019/2020 bzw. die Werte der WS 2021/2022 und 2022/2023 gemittelt. Das WS 2020/2021 wurde ausgeschlossen, da wegen der noch hohen Anrechnung der Wartezeit im Rahmen einer Übergangsregelung eine Verzerrung der Ergebnisse zu erwarten stand.

Spearman-Rangkorrelationen wurden durchgeführt, um für die 16 Bundesländer den Anteil der Eingeschriebenen an den Bewerber:innen mit der mittleren HZB-Note in ein Verhältnis zu setzen.

## Ergebnisse

### Alter, Geschlecht, HZB-Punkte und Berufsausbildung

Um die Veränderung des neuen Zulassungsverfahrens auf die Studierendenschaft zu untersuchen, wurden die Zulassungen der beiden WS 2018/2019 und 2019/2020 (altes Verfahren) mit denen der WS 2021/2022 und 2022/2023 (neues Verfahren) verglichen.

**Figure 2:**
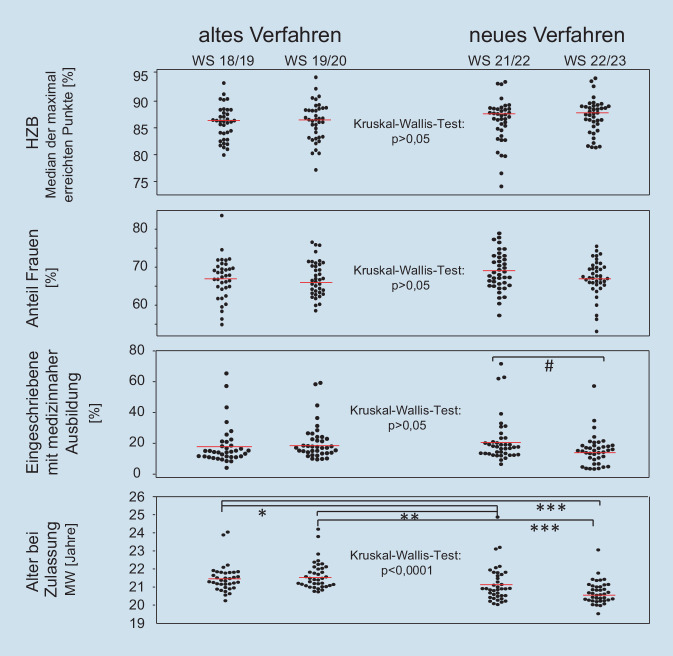

Bezüglich der erreichten HZB-Punkte bei den Eingeschriebenen ergaben sich keine Veränderungen. Abb. 2 zeigt die Mediane der maximal erreichbaren Punkte für die einzelnen Fakultäten. Der nichtparametrische Vergleich der 4 Semester ergab einen *p*-Wert von 0,1326 und deutete damit keine signifikanten Unterschiede an, auch wenn zum WS 2022/2023 der Median leicht angestiegen war. Der Anteil der Frauen an den bundesweit Eingeschriebenen blieb ebenfalls fast konstant, obwohl sich an einzelnen Fakultäten deutliche Veränderungen ergeben haben.

Bei dem Anteil der Eingeschriebenen mit vorheriger medizinnaher Ausbildung ist das Bild noch nicht ganz eindeutig. Bei Einschreibungen nach dem alten Verfahren lag der Anteil bei 17 % bzw. 18 % in den WS 2018/2019 und 2019/2020, im WS 2021/2022 lag er bei 20,5 % und im darauffolgenden Jahr wieder niedriger bei 14,2 %. Der nichtparametrische Vergleich aller 4 Semester resultierte in einem *p*-Wert von 0,1510 und suggeriert damit keine signifikanten Unterschiede. Allerdings zeigt der Vergleich von ausschließlich WS 2021/2022 mit 2022/2023 einen signifikant niedrigeren Anteil (*p* = 0,0405) nach dem Auslaufen der Übergangsregelung und könnte für zukünftige Jahre stehen. Möglicherweise haben viele Ausgebildete die letzte Chance der Anrechnung ihrer Wartezeit im WS 2021/2022 genutzt.

Hochsignifikante Veränderungen gab es beim Alter der eingeschriebenen Studierenden. Hier lag das mittlere Alter vor der Umstellung des Zulassungsverfahrens bei 21,4 bzw. 21,5 Jahren, im WS 2021/2022 bei 21,1 und nach Beendigung der Übergangsphase für die Wartezeit bei 20,6 Jahren.

### Studienort: bevorzugt wohnortsnah

Um zu untersuchen, ob sich die Studierenden bei uneingeschränkter Wahl der Ortspräferenz im neuen System anders über die Republik verteilen, wurden zunächst die jeweiligen Wohnorte bei Bewerbung dem entsprechenden Bundesland zugeordnet und dann analysiert, wo Zulassungen angenommen wurden. Bewerbungen aus dem Ausland blieben hierbei unberücksichtigt.

**Figure 3:**
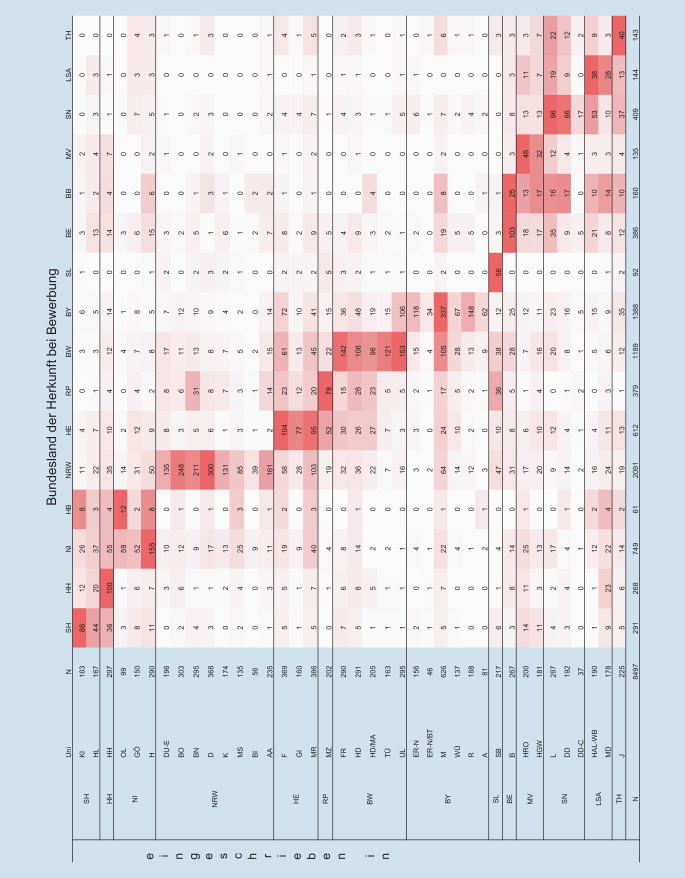

Abb. 3 fasst die Ergebnisse für das WS 2022/2023 zusammen und zeigt in den Spalten, wie viele Studierende aus dem jeweiligen Bundesland sich an welchen Fakultäten eingeschrieben haben. Die Zeilen geben für die einzelnen Fakultäten an, woher die Eingeschriebenen kamen. In der letzten Spalte z. B. sind alle 143 Studierenden mit thüringischer Herkunft gelistet, von denen 40 in Jena studierten, 36 im benachbarten Sachsen und 12 in Sachsen-Anhalt; der Rest verteilte sich in geringen Zahlen über die Republik.

Neben den Eingeschriebenen mit thüringischer Herkunft finden sich in Jena schwerpunktmäßig Studierende aus den Nachbarländern Sachsen, Bayern, Niedersachsen, Hessen und Sachsen-Anhalt. Ähnliche Verteilungsmuster – Einschreibung bevorzugt im Bundesland der Herkunft oder in benachbarten Bundesländern – lassen sich bundesweit und für alle betrachteten Jahrgänge sowohl nach altem und neuem Zulassungsverfahren beobachten.

### Abiturnotenausgleich: mehr Studierende aus „strengen“ Bundesländern

Aufgrund der fehlenden Vergleichbarkeit der HZB-Noten sollte mit dem Länderausgleich die Benachteiligung von Bewerber:innen aus „strengen“ Bundesländern – also Ländern mit im Mittel schlechteren Abiturdurchschnittsnoten – vermieden werden.

Um die Zuteilung der Studienplätze auf die Länder vor und nach Umstellung des Vergabeverfahrens vergleichen zu können, wurden hier die Semester 2018/2019 und 2019/2020 bzw. 2020/2021 und 2021/2022 berücksichtigt, da für das WS 2022/2023 die statistischen Erhebungen zum Zeitpunkt der Manuskripterstellung noch nicht zur Verfügung standen.

**Figure 4:**
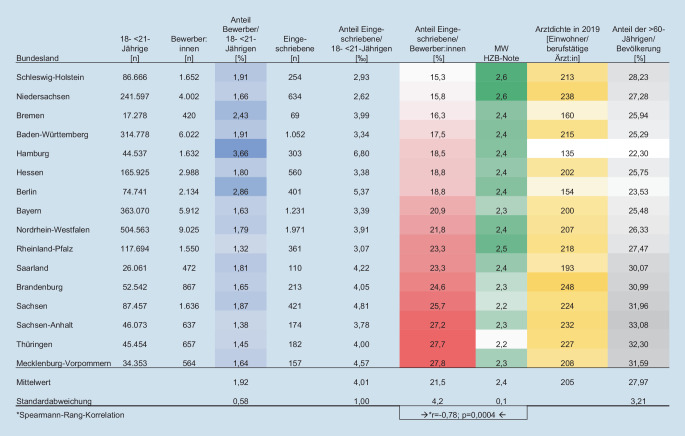

**Figure 5:**
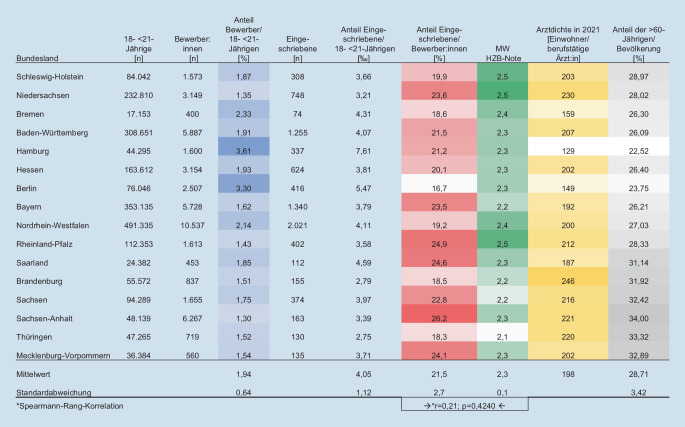

Tatsächlich war unter dem alten Verfahren zu beobachten, dass die Korrelation zwischen dem Anteil der Eingeschriebenen an den Bewerber:innen und dem Landesdurchschnitt der HZB-Noten hochsignifikant und stark negativ war, also Bewerber:innen aus den strengen Bundesländern deutlich seltener einen Studienplatz erhielten (Abb. 4). Durch den Abiturnotenausgleich ist diese Korrelation im neuen Verfahren aufgehoben (Abb. 5). Der Notenausgleichsmechanismus beinhaltet, dass zunächst Landesranglisten der Bewerber:innen aufgrund ihrer HZB-Punktzahlen gebildet und anschließend die 16 Landesranglisten nach dem d’hondtschen Höchstzahlverfahren zu einer Bundesrangliste vereinigt werden [8]. Einen auf diesen Grundlagen beruhenden Ausgleichsmechanismus gab es bereits im alten Auswahlverfahren in der Abiturbestenquote, neu ist die Anwendung im AdH ab dem WS 2020/2021.

Von der Umstellung des Zulassungssystems profitieren Bewerber:innen aus Bundesländern mit schlechteren Abiturdurchschnittsnoten (Abb. 4 und 5). So ist der Anteil der Eingeschriebenen an den Bewerber:innen z. B. in Schleswig-Holstein, einem der „strengsten“ Länder, um 30 % gestiegen. Den gegenteiligen Effekt gab es im „milden“ Bundesland Thüringen mit einer Reduktion um 34 % der Zulassungsquote. Für Niedersachsen mit einem Anstieg von fast 50 % ist die Aussagekraft aufgrund der Rückumstellung von G8 auf G9 im Jahr 2020 stark eingeschränkt.

In den Abiturnotenausgleich gehen zu 2 Drittel der Landesbevölkerungsanteil (Zahl der 18- bis unter 21-Jährigen) und zu einem Drittel der Bewerber:innenanteil (Zahl der Bewerber:innen im Landesbevölkerungsanteil) ein, Stadtstaaten erhalten zusätzlich einen Bonus. Von dieser Regelung profitieren die Stadtstaaten, weil hier bereits der Bewerber:innenanteil am höchsten ist (Mittelwert der WS 2018/2019 und 2019/2020: Hamburg 3,7 %; Berlin 2,9 %). In ländlicher geprägten Bundesländern hingegen ist dieser Anteil niedriger (Rheinland-Pfalz 1,3 %; Thüringen und Sachsen-Anhalt je ca. 1,4 %; Brandenburg und Mecklenburg-Vorpommern je 1,6 %), sodass Studienwillige aus ländlich geprägten Ländern dadurch seltener zugelassen werden.

Insgesamt führt der Abiturnotenausgleich ab dem SS 2020 zu einer Angleichung der Chancen von Bewerber:innen aus unterschiedlichen Bundesländern: Bei einem konstanten Mittelwert von 21,5 % Eingeschriebenen unter den Bewerber:innen sank die Varianz zwischen den Bundesländern von 4,2 % im alten auf 2,7 % im neuen Verfahren. Gleichzeitig führt das neue System dazu, dass in den Stadtstaaten Hamburg und Bremen der Anteil der Eingeschriebenen am Bevölkerungsanteil angestiegen ist.

Im Gegensatz dazu sank er in allen ostdeutschen Flächenländern, wo bessere Abiturnotendurchschnitte mit den geringsten Bewerber:innenanteilen zusammentreffen. In diesen Bundesländern ist daher der Anteil der Eingeschriebenen an der jungen Bevölkerung am niedrigsten, obwohl der Anteil der Eingeschriebenen an den Bewerber:innen hoch ist. Die niedrige Anzahl an Landeskindern, die zu Ärzt:innen ausgebildet werden, trifft in diesen Ländern zudem auf eine geringe Arztdichte und einen hohen Anteil an über 60-Jährigen (Abb. 4 und 5).

## Diskussion und Fazit

Das Urteil des Bundesverfassungsgerichts von 2017 bedingte eine Neuordnung der Studienplatzvergabe in der Humanmedizin, die mit dem SS 2020 von der SfH und den Fakultäten umgesetzt wurde. Das Auswahlkriterium der Wartezeit, das zuletzt bei mindestens 14 Semestern lag, wurde abgeschafft. Daher erstaunt es wenig, dass die Eingeschriebenen im ersten Semester jünger geworden sind. Bezüglich der Geschlechter- und Notenzusammensetzung der Eingeschriebenen zeigten sich insgesamt wenig Veränderungen. An einzelnen Fakultäten jedoch hat sich der Median der HZB-Punkte – am wahrscheinlichsten wegen Art und Gewichtung von Zulassungskriterien – deutlich verändert.

Weil laut Bundesverfassungsgericht die Abiturnote allein keine ausschlaggebende Bedeutung bei der Studienplatzvergabe in der AdH-Quote haben darf, wurde den medizinischen Fakultäten per Staatsvertrag auferlegt, neben der Abiturleistung einen Studieneignungstest plus ein weiteres Auswahlkriterium einzusetzen. Viele Fakultäten nutzen hierbei eine abgeschlossene medizinnahe Ausbildung als Kriterium.

Drei Jahre nach Verfahrensumstellung lässt sich jedoch noch nicht endgültig abschätzen, wie sich der Anteil der Studierenden mit vorheriger medizinnaher Ausbildung entwickelt. Ein hoher Anteil könnte einerseits auf einen verbesserten Hochschulzugang für bisher unterrepräsentierte soziale Gruppen, wie z. B. „first generation students“^1^, hindeuten. Dieses Anliegen wird international unter dem Begriff „widening participation“ [9, 10] diskutiert, zuletzt im Zusammenhang mit dem Urteil des US-Verfassungsgerichts zur „affirmative action“. Ziele sind hier eine Diversifizierung der Ärzteschaft, um Patient:innen aller Kulturkreise optimal zu behandeln [11, 12], sowie ein fairer Studienzugang für Gruppen, die bei den Abiturnoten systematisch benachteiligt sind. Der deutsche Versuch, „widening participation“ über die Zulassung von „ausgebildeten“ Bewerber:innen zu erreichen, kann jedoch im schlimmsten Fall die Abwanderung von qualifizierten Arbeitskräften z. B. aus der Pflege bewirken und somit den dort bereits bestehenden Notstand verstärken [13, 14].

Im alten Zulassungssystem führte die Beschränkung auf 6 Studienortswünsche häufig zu strategisch gewählten Ortspräferenzen, um die Chancen auf einen Studienplatz zu erhöhen. Obwohl im neuen System beliebig viele Ortswünsche in beliebiger Wunschreihenfolge möglich sind, ohne die Chancen an einem anderen Ort zu verschlechtern, wurden die meisten Studierenden nach wie vor einer heimatnahen Universität zugewiesen. Der Wunsch nach heimatnahem Studienort scheint somit unverändert.

Der Bundeslandausgleich der Abiturnoten auch in der AdH-Quote erreicht das angestrebte Ziel, die Zulassungschancen von Bewerber:innen aus Bundesländern mit unterschiedlichem Abiturnotenspektrum anzugleichen. Ein wohl unerwünschter Nebeneffekt durch den Einbezug der Bewerber:innenzahlen ist jedoch, dass dadurch weniger Bewerber:innen aus ländlich geprägten Bundesländern einen Studienplatz erhalten, was zu einer Verstärkung des Landarztmangels beitragen könnte.

So bewerben sich z. B. in Sachsen-Anhalt deutlich weniger junge Menschen – nämlich 1,3 % der 18- bis unter 21-Jährigen – auf einen Medizinstudienplatz, wohingegen die Quote in Hamburg bei 3,6 % liegt. Verglichen mit Sachsen-Anhalt erhält ein mehr als doppelt so hoher Anteil junger Menschen aus Hamburg einen Studienplatz. Vor dem Hintergrund, dass sich Ärzt:innen, die aus ländlichen Gebieten stammen oder dort einen Teil ihrer Ausbildung absolviert haben, auch am ehesten in ländlichen Gebieten niederlassen [15, 16], scheint der aktuelle Abiturnotenausgleichsmechanismus zwar fair für Bewerber:innen aus unterschiedlichen Bundesländern zu sein, könnte aber gleichzeitig das Problem des Landarztmangels verschärfen.

Natürlich gibt es neben der Herkunft auch andere Einflussfaktoren auf den Ort der Tätigkeit nach Abschluss des Medizinstudiums, wie z. B. eine Bindung an den Studienort oder die Vergütung/Anzahl der Privatpatient:innen am Tätigkeitsort. Dennoch zeigen die Studien, dass die Herkunft aus einer ländlichen Region der beste Prädiktor für eine ärztliche Tätigkeit in einer ländlichen Region ist.

Vermutlich wird sich auch in Bundesländern mit beidem, großen Städten und ländlichen Regionen, ein deutlich höherer Anteil der Städter für ein Medizinstudium bewerben und nach dem Studium dann auch in den Städten praktizieren. Städte übernehmen zwar eine Versorgungsfunktion für angrenzende ländliche Regionen, dennoch ist vor allem die Gesundheit der Bewohner:innen ländlicher Regionen durch den Ärztemangel gefährdet. Aktuell sind Ärzt:innen in Sachsen-Anhalt für fast doppelt so viele Patient:innen verantwortlich wie in Hamburg, obwohl die ländliche Bevölkerung älter ist als die städtische – der Anteil der über 60-Jährigen liegt in Sachsen-Anhalt bei 34 %, in Hamburg dagegen nur bei 22,5 % (Abb. 5) – und alte Menschen häufiger ärztliche Hilfe benötigen [17].

Viele Bundesländer versuchen mit der Einführung der Landarztquote dem Landarztmangel entgegenzusteuern; deren Erfolg ist bisher allerdings noch nicht absehbar. Zusätzlich könnte eine Veränderung des Abiturausgleichsmechanismus zur Behebung des Landarztmangels beitragen: Wenn, wie ursprünglich schon vorgeschlagen [18], der Ausgleichsmechanismus nur auf der Anzahl der 18- bis unter 21-Jährigen in einem Bundesland beruhte, ohne die Bewerber:innenzahl oder Boni für Stadtstaaten zu berücksichtigen, würde die Zahl der angehenden Mediziner:innen aus einem Bundesland besser an den regionalen Bedarf angepasst. Noch weitreichender wäre die Anpassung, wenn der Ausgleichsmechanismus auf dem Anteil der über 60-Jährigen beruhte. Schwieriger wird es, über den Abiturausgleichsmechanismus innerhalb eines Bundeslandes stärker Bewerber:innen aus ländlichen Regionen zu berücksichtigen, z. B. über eine Berücksichtigung der Bevölkerungszahl einzelner Landkreise.

Um auf einem dieser Wege einer möglichen medizinischen Unterversorgung in ländlichen Regionen zu begegnen, müssten die Bundesländer den Staatsvertrag zur Hochschulzulassung entsprechend modifizieren.

## Footnotes

1 Studierende, deren Eltern keinen Hochschulabschluss haben.
